# Whole Blood Transcriptomics in Cardiac Surgery Identifies a Gene Regulatory Network Connecting Ischemia Reperfusion with Systemic Inflammation

**DOI:** 10.1371/journal.pone.0013658

**Published:** 2010-10-27

**Authors:** Orfeas Liangos, Sophie Domhan, Christian Schwager, Martin Zeier, Peter E. Huber, Francesco Addabbo, Michael S. Goligorsky, Lynn Hlatky, Bertrand L. Jaber, Amir Abdollahi

**Affiliations:** 1 The Kidney and Dialysis Research Laboratory, Division of Nephrology, Caritas St. Elizabeth's Medical Center, Tufts University School of Medicine, Boston, Massachusetts, United States of America; 2 Center of Cancer Systems Biology, Caritas St. Elizabeth's Medical Center, Tufts University School of Medicine, Boston, Massachusetts, United States of America; 3 Department of Nephrology, University Medical School and German Cancer Research Center, Heidelberg, Germany; 4 Department of Radiation Oncology, University Medical School and German Cancer Research Center, Heidelberg, Germany; 5 Division of Nephrology, New York Medical College, Valhalla, New York, United States of America; National Cancer Institute at Frederick, United States of America

## Abstract

**Background:**

Cardiac surgery with cardiopulmonary bypass (CS/CPB) is associated with increased risk for postoperative complications causing substantial morbidity and mortality. To identify the molecular mechanisms underlying CS/CPB-induced pathophysiology we employed an integrative systems biology approach using the whole blood transcriptome as the sentinel organ.

**Methodology/Principal Findings:**

Total RNA was isolated and globin mRNA depleted from whole blood samples prospectively collected from 10 patients at time points prior (0), 2 and 24 hours following CS/CPB. Genome-wide transcriptional analysis revealed differential expression of 610 genes after CS/CPB (p<0.01). Among the 375 CS/CPB-upregulated genes, we found a gene-regulatory network consisting of 50 genes, reminiscent of activation of a coordinated genetic program triggered by CS/CPB. Intriguingly, the highly connected *hub nodes* of the identified network included key sensors of ischemia-reperfusion (HIF-1alpha and C/EBPbeta). Activation of this network initiated a concerted inflammatory response via upregulation of TLR-4/5, IL1R2/IL1RAP, IL6, IL18/IL18R1/IL18RAP, MMP9, HGF/HGFR, CalgranulinA/B, and coagulation factors F5/F12 among others. Differential regulation of 13 candidate genes including novel, not hitherto CS/CBP-associated genes, such as PTX3, PGK1 and Resistin, was confirmed using real-time quantitative RT-PCR. In support of the mRNA data, differential expression of MMP9, MIP1alpha and MIP1beta plasma proteins was further confirmed in 34 additional patients.

**Conclusions:**

Analysis of blood transcriptome uncovered critical signaling pathways governing the CS/CPB-induced pathophysiology. The molecular signaling underlying ischemia reperfusion and inflammatory response is highly intertwined and includes pro-inflammatory as well as cardioprotective elements. The herein identified candidate genes and pathways may provide promising prognostic biomarker and therapeutic targets.

## Introduction

Cardiac surgery including coronary artery bypass grafting (CABG) and valvular replacement or repair are among the most frequently performed life-saving surgical procedures. Since the introduction of cardiopulmonary bypass (CPB) in the 1950s, extracorporeal circulation became an integral component of cardiac surgery [1]. Despite continuous technical improvements, cardiac surgery with CPB (CS/CPB) provokes tissue ischemia-reperfusion injury and a vigorous systemic inflammatory response that may ultimately lead to the development of postoperative complications [2], [3]. A frequent complication of this CS/CPB-induced systemic inflammatory response syndrome (SIRS) is the development of organ dysfunction, including the lungs and kidneys [4], [5]. It has been reported that 20% of “low risk” patients who undergo CS/CPB may develop postoperative complications [6] and the incidence of multiple organ dysfunction syndrome (MODS) was 11% with mortality rates of 41% for this group of patients [7]. Despite these morbidity and mortality rates little is known about the molecular mechanisms underlying the pathophysiology of CS/CPB-induced postoperative complications.

The surgical trauma, blood loss, transfusion of blood products and hypothermia were discussed as non specific stimulants of the cardiac surgery-induced inflammatory response [8]. However, the CS/CPB-induced pathophysiology appears to be a composite of at least two distinct mechanisms that specifically contribute to the inflammatory response. First as a result of the aortic cross-clamping, unstable peri-bypass hemodynamics, global myocardial ischemia and suboptimal organ perfusion during CPB ischemia–reperfusion injury may occur in various organs, including the brain, heart, lung, kidney, intestine and liver. A second critical component of the immune response might be the direct “contact activation” (priming) of the circulating blood leukocytes *via* exposure to the foreign surfaces of the extracorporeal CPB circuit [8]–[19].

Research in this area has thus far focused predominantly on quantifying and correlating the levels of pertinent circulating inflammatory proteins with exposure to the CS/CPB event or with clinical outcomes. Further, differential regulation of genes and proteins in ischemic organs as well as in the blood during the ischemia-reperfusion process have been studied [20]–[22]. These studies were focused on identifying differential regulation of genes during CS/CPB as a function of disease states, e.g. vascular permeability in patients with diabetes mellitus, or in association with clinical outcomes such as neurocognitive decline or atrial fibrillation. These correlative data have improved our understanding of the CS/CPB-induced inflammatory response. Nonetheless, a systematic and integrative analysis of the molecular mechanisms underlying the initiation and execution of the inflammatory response following CS/CPB is still needed.

In contrast to previous approaches as outlined above, we sought to investigate the circulating blood cell transcriptome as a sentinel organ that is exposed to both incriminated mechanisms, direct contact activation *via* the extracorporeal membrane of the CPB circuit as well as ischemia-reperfusion mechanisms occurring in the heart, lungs and other organs during cardiac surgery. We hypothesized that the exposure of circulating blood cells, including leukocytes and leukocyte progenitor cells, to these stimuli will result in a perturbation of their transcriptome enabling the detection of the sensing event and the molecular signaling that governs the CS/CPB-induced systemic inflammatory response.

## Methods

### Study design and patient selection

This is an ancillary prospective cohort study conducted between February 2006 and January 2007 at St. Elizabeth's Medical Center in Boston, MA, as part of an ongoing parent study aimed at evaluating genetic risk markers for acute kidney injury following cardiac surgery. All consecutive adult subjects (age 18 years or greater) scheduled to undergo on-pump cardiac surgery were eligible for enrollment. Exclusion criteria were age under 18 years, off-pump surgery, pregnancy, long-term or acute dialysis, and organ transplantation within the prior year. Written informed consent was obtained from all study participants or next of kin. The Research/Human Subjects Committee of the institutional review board at St. Elizabeth's Medical Center approved the study protocol, and the study was conducted in accordance with the ethical standards laid down in the Declaration of Helsinki of 1975, as revised in 1983.

For the purpose of the whole blood genome-wide transcriptional analysis, out of the parent study, we selected ten patients sharing similar pre-operative clinical characteristics including age, preoperative serum creatinine, left ventricular ejection fraction, inclusion of valvular surgery and CPB perfusion time.

For the purpose of plasma protein analyses, as a confirmatory selected gene product analysis, 34 additional patients undergoing cardiac surgery were identified from the parent study.

### Data collection

Medical records of study participants were reviewed prospectively to retrieve pre-operative variables including baseline demographic characteristics, coexisting conditions, intraoperative variables including CPB time, cross-clamp time, surgery type, and post-operative variables including serial serum creatinine values and hospital outcomes.

### Blood sampling

For peripheral blood RNA sampling, venous whole blood was collected immediately before, and 2 and 24 hours following CPB into PAXgene Blood RNA collection tubes (PreAnalytiX GmbH, Hombrechtikon, CH), which were initially stored at −20°C until extraction, and then stored at −80°C.

For plasma protein analysis, EDTA-anticoagulated blood was collected immediately before and at 2, 24 and 48 hours following CPB. Samples were kept on ice and processed within 30 minutes of collection. Plasma was separated immediately by double centrifugation at 3000 RPM for 5 minutes and 10,000 RPM for another 5 minutes, to remove platelets, each at +4°C. Plasma was immediately transferred and the samples stored at −80°C.

### RNA isolation, globin mRNA depletion, and RNA integrity assessment

Whole blood total RNA was extracted using the PAXgene blood RNA extraction kit (Qiagen, Hilden, Germany). Globin mRNA is expressed at high levels in erythrocytes and reticulocytes, and up to 70% of the mRNA in whole blood total RNA consists of globin transcripts, which decreases the sensitivity of blood-based expression profiling techniques for detecting less abundant mRNAs [20]–[23]. This reduced sensitivity might have limited previous whole blood transcriptomic approaches to detect low-copy number genes, e.g. using inflammatory genes tailored microarrays [24]. To enhance the overall sensitivity of the expression profiling platform and to detect low-abundance mRNAs, α and β globin mRNAs were depleted from total RNA by selective hybridization and magnetic bead separation method, using the GLOBINclear Kit (Ambion, Austin, TX). The RNA quality was assured after both Paxgene total-RNA isolation and globin mRNA depletion by assessing the 18S/28S ratio and RNA-integrity (RIN) scores. RNA integrity was assessed by lab-on chip technology, using the Agilent 2100 bioanalyzer, in combination with the RNA 6000 Lab Chip kit (Agilent Technologies, Boeblingen, Germany). All kits were used according to the manufacturer's instructions. RNA quantity was measured using the NanoDrop™ Spectrophotometer (Thermo Scientific, Waltham, MA).

### Genome-wide expression profiling

Genome- wide expression profiling was performed using *whole human genome 4x44k oligo microarrays* (Agilent, G4112F). Linear amplification from 500 ng of total RNA and spike-in-controls (Agilent #5188–5282) was performed using the Agilent *low RNA Input Linear Amplification Kit Plus*, *one color* (#5188–5339). During this amplification, direct labelling of the probe was performed by incorporation of fluorescently labelled nucleotides into the amplified RNA. Labelled probes were purified with *RNeasy* mini spin columns (Qiagen #74104). Cy3- labelled probe and blocking agent were combined for chemical fragmentation of the probe molecules. Fragmentation was stopped by adding 55 µl 2x GEx hybridisation buffer HI-RPM leading to a final volume of 110 µl (Gene expression hybridization kit, Agilent #5188–5242). Chip hybridization was performed using the gasket/slide-sandwich system and the hybridisation chamber (Agilent, #G2534A) according to manufacturer instructions. During hybridisation slides were rotated at 10 rpm and 65°C for 16 h. Microarray slides were washed 1 min in GE Wash Buffer 1 (Agilent, #5188–5326) at room temperature, 1 min in GE Wash Buffer 2 (Agilent, #5188–5326) at room temperature and 30s in Acetonitril at room temperature on a magnetic stirrer. Microarray slides were scanned in an Agilent Microarray Scanner and analysis of the resulting array images was performed using the *Feature Extraction* software (Agilent, Version 9.1). Microarray data were stored in the MO-MEX database Bloader, which enables direct submission of large batches of MIAME compliant expression profiling data to the ArrayExpress database. Microarray data are available online at ArrayExpress (http://www.ebi.ac.uk/arrayexpress), under the accession number E-TABM-595.

### Microarray data analysis

Generation of expression matrices, data annotation, filtering and processing were performed as described previously using the TableButler software package (http://www.OncoExpress.org/software/tablebutler) [25], [26]. All microarray statistics including *t*-test and analysis of variance (ANOVA) with permutation analysis (n = 1000) and cluster analysis were performed using the SUMO software package [25]–[27]. Pathway analysis was performed based on information available on cellular signaling processes (e.g. protein-protein interaction, geneontology, involvement in specific signaling pathway etc.) using a curated database on signaling networks and systems biology package (Metacore®, Genego, www.genego.com). The metacore manually annotated database was derived from literature publications on proteins and small molecules (MetaCore™, GeneGo, St. Joseph, MI). This was developed with an Oracle version 9.2.0.4 Standard Edition (Oracle, Redwood Shores, CA) based-architecture for the representation of biological functionality and integration of functional, molecular, or clinical information. To construct the network, we searched and analyzed direct interactions among the CPB-induced genes in the whole blood transcriptome.

### Real-time quantitative RT-PCR

Expression levels of RNA transcripts were quantitated by the ABI 7300 real time PCR system (Applied Biosystems, Foster City, CA) as previously described [25], [28], [29]. After RNA isolation, first strand cDNA was reverse transcribed from total RNA using the cDNA Archive Kit (MultiScribe Reverse Transcriptase, Applied Biosystems) and stored at −20°C until use. Complementary DNAs were mixed with PCR master mix and primers (Applied Biosystems), and real-time PCR was performed. TaqMan® primers and probes information is listed in Appendix S1.

In addition to profiling all samples for the target sequence, samples were profiled for 18S ribosomal RNA expression as an endogenous control. For low copy number genes, β macroglobulin was used as an alternative endogenous control. For each single well amplification reaction, a threshold cycle was observed in the exponential phase of amplification and the quantitation of relative expression levels was achieved using standard curves for both the target and endogenous controls.

### Plasma protein analyses

Plasma levels of three representative proteins, matrix metalloproteinase-9 (MMP-9), macrophage inflammatory protein-1 alpha (MIP-1α) and macrophage inflammatory protein-1 beta (MIP-1β), found to be upregulated by genome-wide expression profiling, were measured by the Luminex microbeads technique (Linco Inc., St. Louis, MO). In brief, 25 µl of sample, standard solutions or quality control samples were added to each well of a 96-well plate with 25 µl of the bead solution. The plate was incubated overnight on a plate shaker at 4°C, washed twice with 200 µl each of wash buffer, removing buffer by vacuum filtration between each wash, followed by addition of 25 µl of a detection antibody cocktail and incubated at room temperature for 1.5 h. After adding 25 µl of a streptavidin-phycoerythrin solution to each well and incubating at room temperature for 30 min, the plates were then analyzed on the LuminexIS100 analyzer (Luminex Inc., Austin TX). The data were evaluated as Median Fluorescence Intensity (MFI) using appropriate curve-fitting software (Luminex 100IS software version 2.3). All measurements were performed in duplicate. Analyses were performed in duplicates and the responsible investigators (Francesco Adabbo, PhD and Michael Goligorksy, MD, PhD) were blinded to the results of the genome-wide expression profiling and RT-PCR.

### Statistical analyses

Continuous variables are presented as means (± standard deviation), and categorical variables as frequencies. For the microarray data, clustering and statistical analysis were performed using SUMO and Genego (metacore) software packages. Multiple comparisons were performed using the Bonferroni method, and differences were considered statistically significant at a P value of less than 0.05.

For the plasma protein analysis, after log-transformation of the data due to skewed distribution, Kruskal-Wallis test for change over time was used to compare between 4 measurement time points (pre-CPB, and 2-hour, 24-hour and 48-hour post-CPB). These statistical analyses were performed using SAS software (version 9.1; SAS Institute, Cary, NC). Differences were considered statistically significant at a P value of less than 0.05.

## Results

### Characteristics of the cohort

The clinical characteristics of each of 10 study participants selected to detect perturbations of the whole blood transcriptome in response to cardiac surgery with CPB are shown in Table 1. In brief, patients were predominantly male with a mean age of 70±11 years, all of whom had hypertension. The left ventricular function was preserved with a mean ejection fraction of 58±10%, and the kidney function was normal with a mean serum creatinine of 0.98±0.18 mg/dl. 50% of the patients underwent valvular surgery, and CPB perfusion time averaged 121±26 min.

**Table 1:** Clinical characteristics of the study cohort.

Patient ID	244	246	247	252	253	259	261	262	268	271
Age (years)	61	69	82	79	62	74	53	57	80	80
Sex	M	M	M	M	F	M	M	M	M	M
Hypertension	Yes	Yes	Yes	Yes	Yes	Yes	Yes	Yes	Yes	Yes
Chronic lung disease	No	No	Yes	No	No	No	Yes	No	Yes	No
Congestive heart failure	No	No	No	No	Yes	No	No	No	No	Yes
Diabetes mellitus	No	No	No	No	Yes	No	No	No	No	No
Left ventricular ejection fraction (%)	ND	55	65	55	ND	65	35	65	60	60
Baseline serum creatinine (mg/dl)	1.0	1.0	0.8	0.9	1.4	1.0	0.9	1.1	0.9	0.8
Cardiopulmonary bypass time (min)	85	174	98	120	137	134	98	104	133	127
Valve surgery	No	No	Aortic	Mitral	Aortic	No	No	No	Aortic	Aortic

ND denotes not determined.

### Genome-wide transcriptional analysis

**Figure 1:**
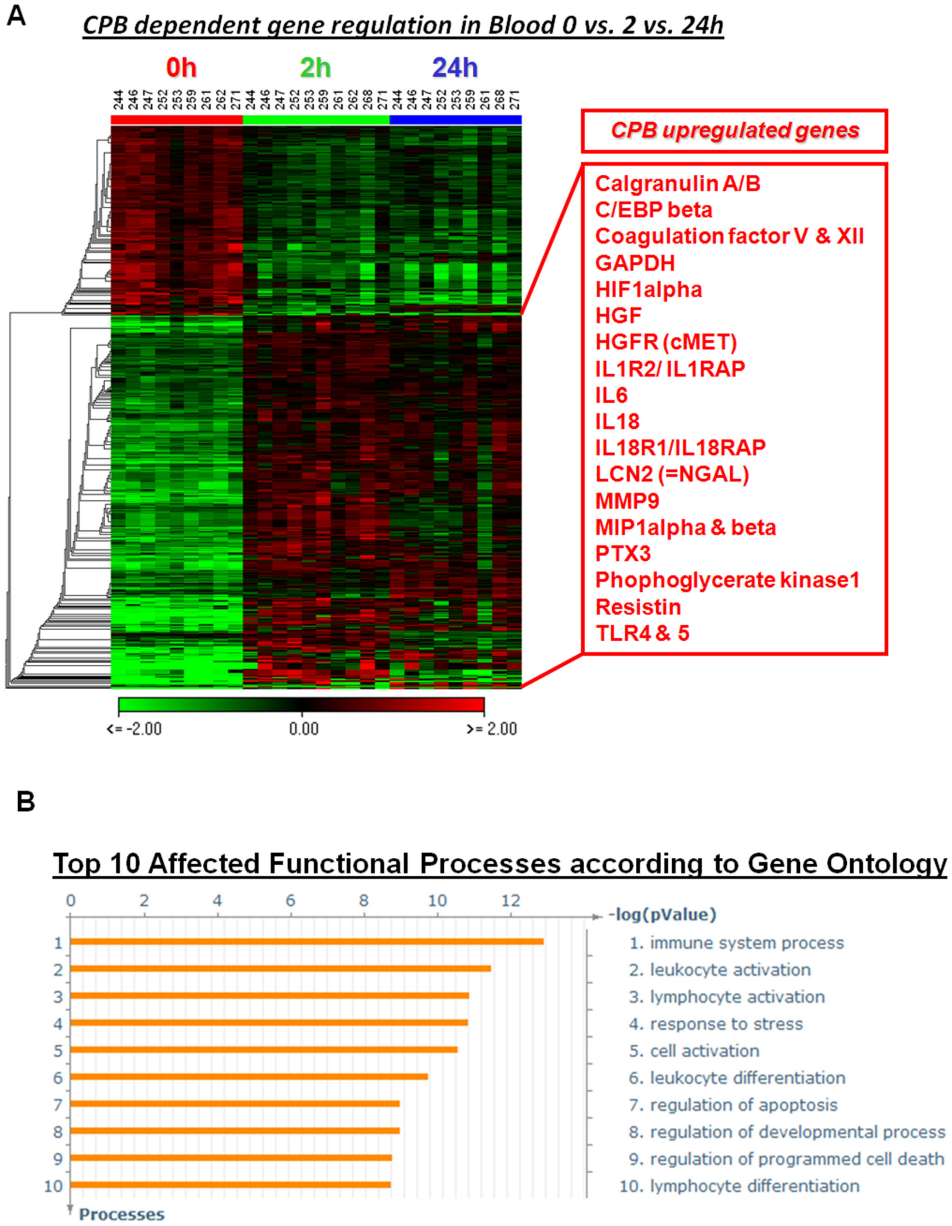
Genome-wide expression profiling. ***Whole blood gene expression profiles*** (***Panel A***). Whole blood cell expression profiles before (0), 2 h and 24 h post CPB revealed 916 differentially regulated transcripts corresponding to 610 known genes (p<0.01). The selected sets of genes are presented as a heatmap using hierarchical clustering (HCL) with Euclidean distances and complete linkage analysis (for a detailed gene list see supplemental SOM table S1). Each row represents lg2 expression ratios of an individual gene (CS/CPB vs. average intensity of all measurements) and the columns indicate each respective patient (i.e., sample time points). Expression ratios are colored according to the scale bar: green  =  downregulation, red  =  upregulation. Representative CS/CPB-induced genes are highlighted in the red box. Selected CS/CPB-induced genes are highlighted in the red box. ***Functional processes*** (***Panel B***). The top 10 functional processes affected by CS/CPB treatment are presented. Data analysis among CS/CPB regulated genes from the selected gene set resulted in significant enrichment for gene ontology processes and cell survival or death. Bars represent -log p-values representing the probability for the gene ontology mapping arising by chance.

In total, 28 blood samples that passed the RNA quality criteria (RIN>7) were hybridized against pan genomic (44K) oligo-microarrays. Gene intensities were quintile and virtual pool normalized. The expression ratios were lg2 transformed. Genes with an average intensity of less than 10 over all samples were removed. We found 6,351 transcripts to be differentially regulated from the time point prior to CPB (0 hour) *vs.* 2 hours *vs.* 24 hours post-CPB (P<0.01 by ANOVA). To most stringently select for candidate CS/CPB-regulated genes, we applied multiple testing correction, and identified 916 differentially regulated transcripts (P<0.01, after standard Bonferroni correction). The 916 transcripts represented 610 unique, i.e., known genes, among which 375 genes were upregulated, whereas 235 genes were downregulated after CS/CPB (Figure 1A). The selected sets of genes are presented as a heatmap using hierarchical clustering (HCL) with Euclidean distances and complete linkage analysis. A detailed list of the significantly regulated genes is presented in supplemental SOM Table S1. To identify the critical involvement of specific functional gene categories in response to CS/CPB, we mapped the 610 CS/CPB-regulated genes to the corresponding *gene ontologies* according to the information available on www.geneontology.org. The top 10 significantly affected functional processes by CS/CPB were selected and sorted based on the lowest p-values (Figure 1B). We found an enrichment of functional gene categories related to the immune system processes such as leukocyte activation and differentiation, and cell survival/apoptosis signaling among the CS/CPB regulated genes. These data demonstrate the feasibility of whole blood transcriptome analysis to uncover the molecular mechanisms underlying the inflammatory response evoked by CS/CPB.

### Identification of a CS/CPB–induced transcriptional network

**Figure 2:**
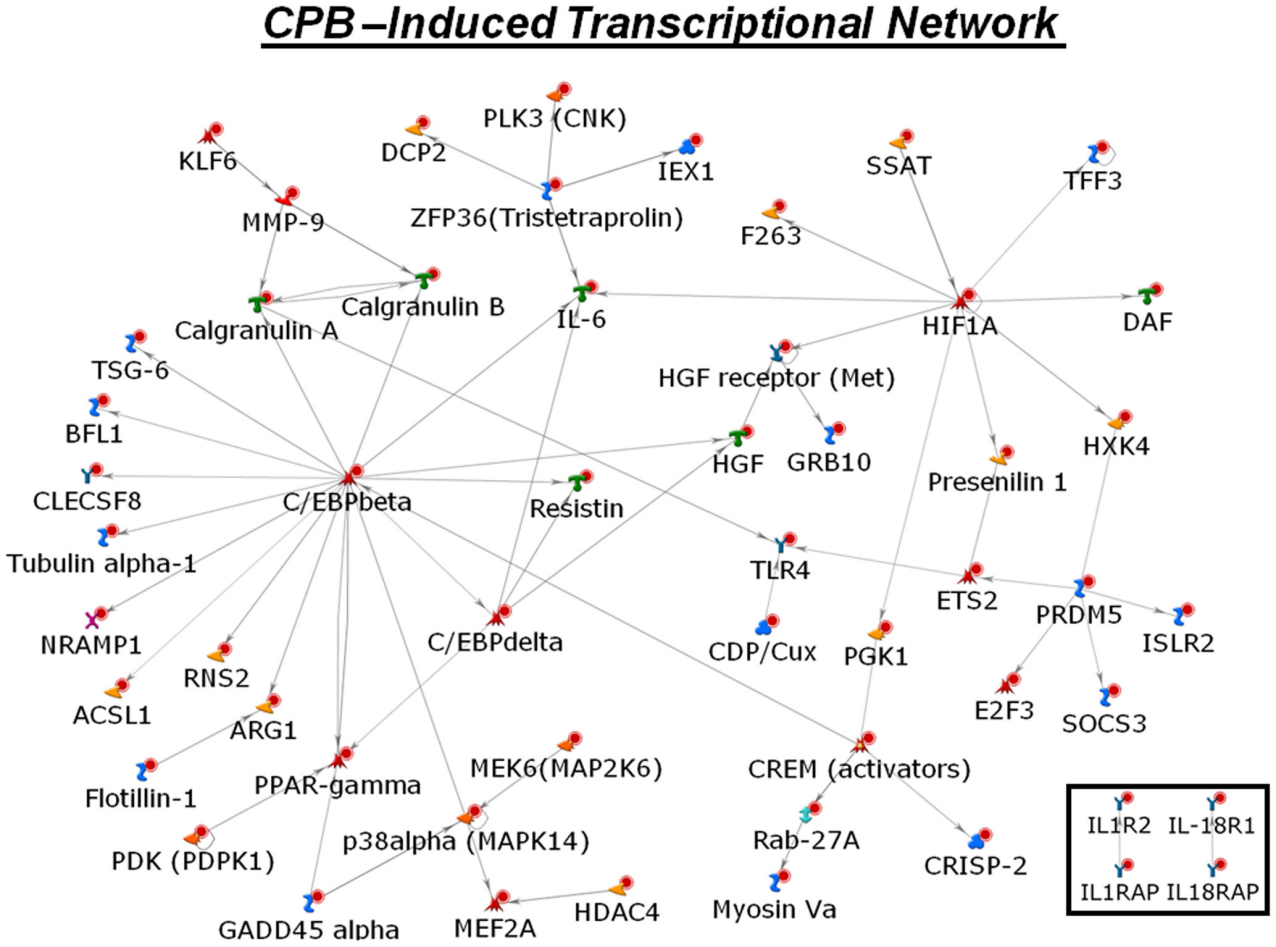
CS/CPB-induced gene regulatory network. A substantial fraction of CS/CPB-induced genes including both hypoxia and inflammatory related genes are directly connected and constitute an intricate transcriptional network. Network analysis reveals a critical role for hypoxia sensors HIF1alpha and c/EBPbeta in CS/CPB response. These data suggest that gene regulatory networks rather than single pathways govern the hypoxia- and immune-modulatory effects triggered by CS/CPB. Four members of IL1 receptor family were also upregulated after CS/CPB (small box).

To analyze the microarray data in an integrative manner, we searched for potential known interactions among the herein identified CS/CPB-induced genes. Intriguingly, we found that a substantial fraction of CS/CPB upregulated genes interact directly with each other and constitute an intricate gene regulatory network (Figure 2). Our data indicate that a coordinated genetic program rather than single pathways governs the inflammatory response evoked by the cardiac surgery with CPB. Interestingly, the high degree or *hub nodes* of the network consisted of known key hypoxia sensors such as the transcription factor hypoxia-inducible factor 1 alpha (HIF1alpha) and CCAAT/Enhancer binding protein (C/EBPbeta). Of note, the involvement of C/EBPbeta in myocardial ischemia reperfusion injury was only recently described in an experimental rat model [30]. The activation of C/EBPbeta was attributed to the transcriptional upregulation of the key proinflammatory protein interleukin 6 (IL-6). Our network analysis revealed that in addition to IL-6, C/EBPbeta is directly regulating at least 15 other CS/CPB-induced genes, including prominent inflammatory response elements such as *Calgranulin* A and B (also known as Leukocyte L1 antigen complex) or *Resistin*. Conclusively, the network analysis connects mechanisms of ischemia-reperfusion injury with systemic inflammatory response mechanisms, and suggests a key role for tissue ischemia in direct upregulation of a plethora of inflammatory associated genes such as matrix metallaproteinases (MMP9), and Toll-like receptors (TLR4/5). We also found four members of the interleukin 1 (IL-1) receptor family, i.e., IL-1 receptor 2 (IL-1R2), IL-1 receptor associated protein (IL-1RAP), IL-18R1 and IL-18RAP, to be upregulated in response to CS/CPB (box in Figure 2).

### Confirmatory real time quantitative RT PCR analysis

**Figure 3:**
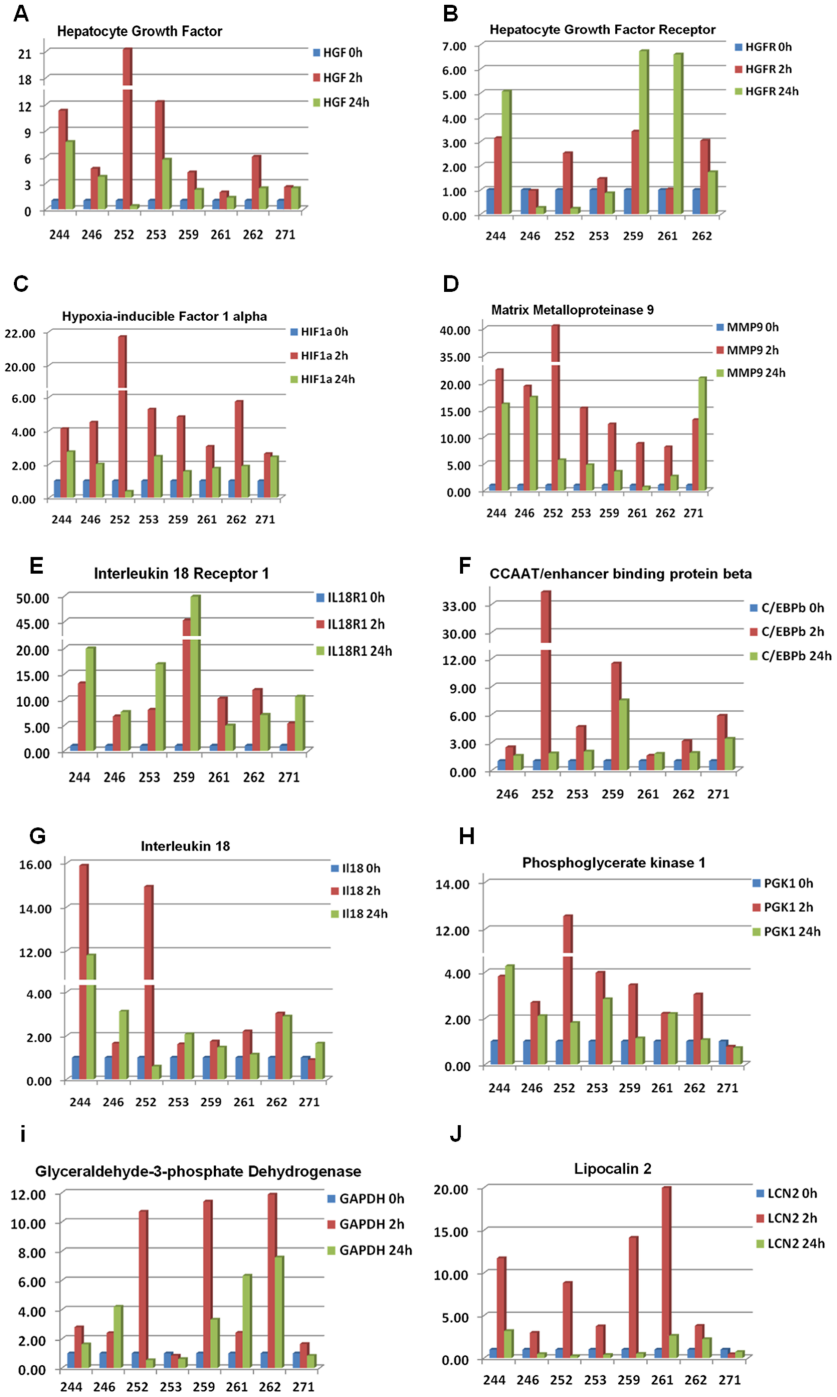
Confirmatory RT PCR analysis of selected genes. Bar graphs show fold-increases in mRNA of one gene, named at the top of each figure. Each respective time point is represented by a different color: blue for the pre CPB (0 h), red for the 2 hour post CPB and green for the 24 hour post CBP time point.

**Figure 4:**
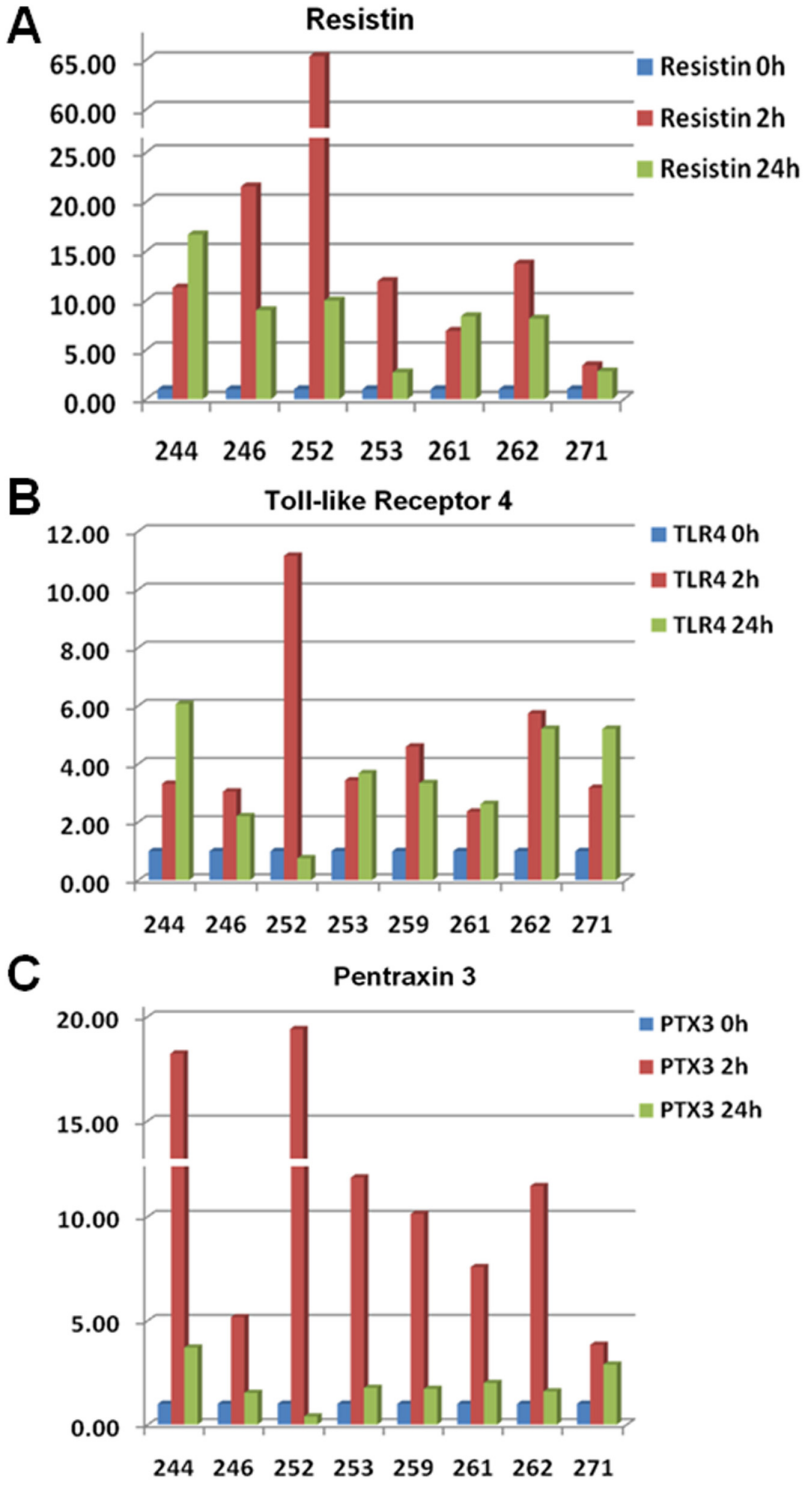
Resistin, PTX3 and TLR4 mRNA expression levels. The mRNA expression level of CS/CPB -induced genes detected by real time qPCR parallels the microarray data.

To confirm the differential regulation pattern of genes observed by microarray analysis after CS/CPB, we selected 13 genes for confirmation analysis by real time quantitative RT-PCR (qPCR). Relative expression levels of genes (compared to the pre-CPB time point) are presented in Figures 3 and 4. The regulation of both *hub nodes* of the CS/CPB–induced network, HIF1alpha and C/EBPbeta, was confirmed by qPCR. Both genes were consistently upregulated in all patients with a maximum peak 2 hours post-CPB. The expression of their downstream network targets such as the Hepatocyte Growth Factor/Receptor pair (HGF/HGFR), Toll-like receptor 4 (TLR4), *Resistin* and MMP9, was also confirmed. From the IL-1 receptor family, the CS/CPB–induced enhanced expression of IL-18R1 and its ligand IL-18 was detected. Further, the expression of four genes that were significantly upregulated after CS/CPB in the microarray analysis (P<0.01, supplemental SOM Table S1) but not included in the direct interaction network, was also confirmed by qPCR. These included genes involved in oxidative stress response, such as Glyceraldehyde-3 Phosphate Dehydrogenase (GAPDH), novel early predictors of organ dysfunction, e.g. Lipocalin 2 (LCN2, also known as NGAL) and glucose metabolizing enzyme phosphoglycerate kinase 1 (PGK1), and a novel gene that is proposed to exert potent cardioprotective effects, Pentraxin 3 (PTX3).

### Confirmatory serum protein analysis

**Figure 5:**
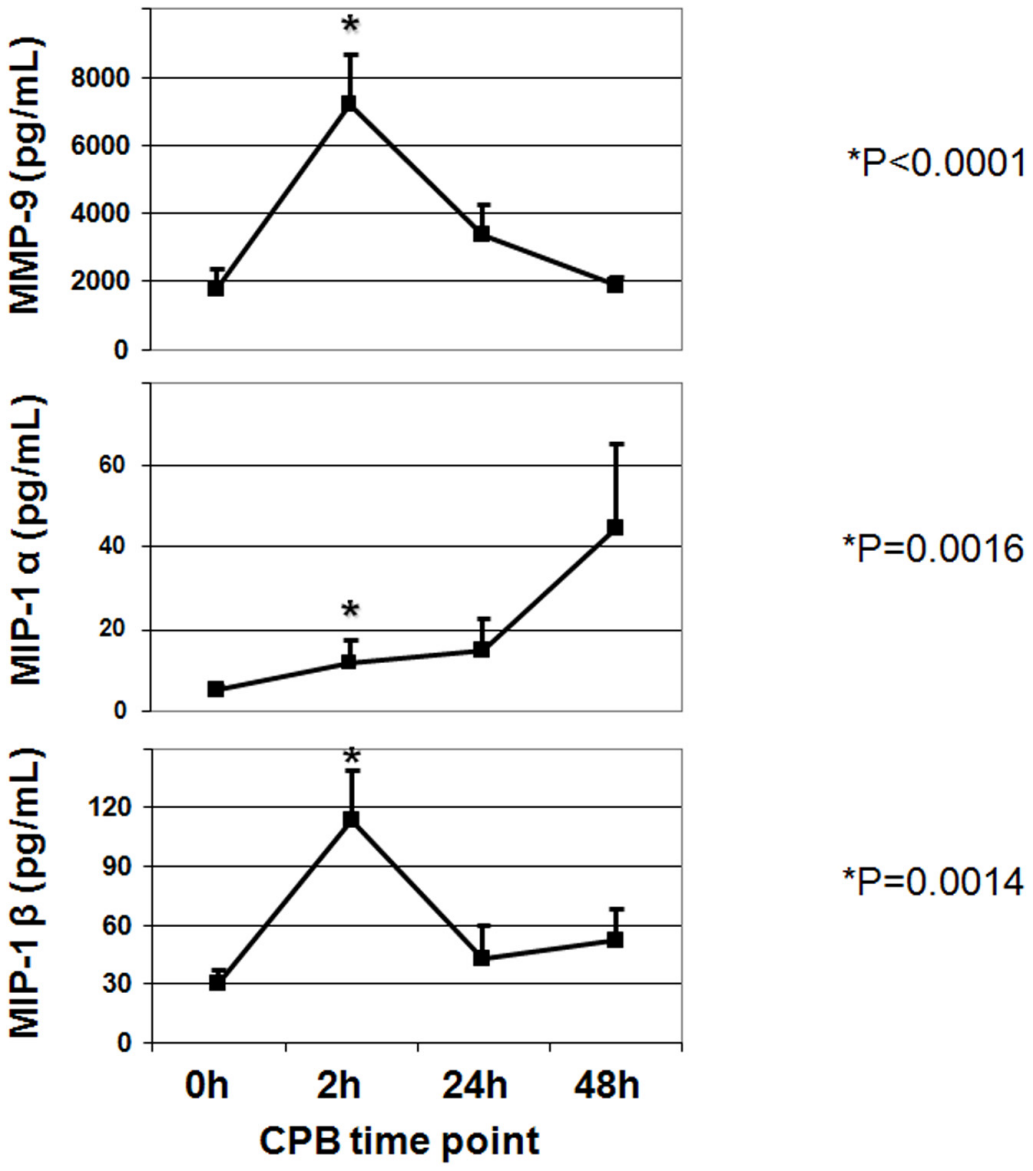
Plasma protein level confirmation. Plasma protein levels (pg/mL) of three CS/CPB –induced genes identified by genome -wide expression profiling. Protein analysis confirmed the whole blood transcriptomics data. We found a time dependent upregulation of matrix metalloproteinase-9 (MMP9) and macrophage inflammatory protein-1 α and β (MIP1a/b) after CS/CPB in an independent cohort of additional 34 patients undergoing on-pump cardiac surgery. Mean (standard error of the mean). * P values; Kruskal-Wallis test for change over time.

To confirm the differential regulation pattern of genes on protein level, plasma samples of 34 additional patients undergoing on-pump cardiac surgery were analyzed. Subjects in this group had a mean age of 72±11 years, a left ventricular ejection fraction of 51±15%, a preoperative serum creatinine of 1.1±0.3 mg/dl, and underwent CPB perfusion for 132±42 minutes. 75% were male, and 71% had hypertension. In support of the mRNA data, we detected a CS/CPB and time dependent increase in MMP9, MIP1α and MIP1β protein levels (Figure 5). While plasma MIP1α demonstrated a linear increase over time post-CPB, both MMP9 and MIP1β reached their maximum concentration 2 hours post-CPB. Together, these data indicate that the regulation of genes detected by whole blood transcriptomics was translated in differential expression of the corresponding proteins in circulating blood.

## Discussion

Our data indicate that blood cells, including circulating leukocytes and bone marrow derived progenitor cells, are dynamic participants in the pathophysiology of CS/CPB-induced host inflammatory response. Using circulating blood cells and their transcriptome as the sentinel organ, we have devised a novel strategy to decipher the molecular mechanisms underlying CS/CPB–induced tissue ischemia-reperfusion injury and systemic inflammatory response. In contrast to plasma or serum protein profiles that provide associative rather than causative information (e.g. potential stimuli or the source of the secreted proteins remain undetermined), the whole blood transcriptomics provided a unique opportunity to causally link the transcriptional perturbation induced by exposure of circulating leukocytes to tissue ischemia or the extracorporeal CPB membrane with the execution of the inflammatory response.

The microarray data were rigorously validated by confirming the differential mRNA expression of 13 CS/CPB-induced genes by real time quantitative RT-PCR. To demonstrate that the mRNA expression levels were indeed translated in differential protein levels, we confirmed the protein expression of three candidate CS/CPB-induced genes, i.e., MMP9, MIP1α and MIP1β in an independent cohort of 34 additional patients.

The advantage of our genome-wide transcriptional analysis over single protein or tailored pathway specific approaches was to further the ability of analyzing the data in an integrative manner and to detect system level convergence of pathways that were previously believed to be distinct. By applying this approach to CS/CPB-induced genes in peripheral blood, we identified a gene-regulatory network connecting the signals mediated by exposure of circulating blood cells to ischemia and reperfusion in heart, lungs as well as distant organs (e.g. HIF1apha) or priming of leukocytes via the extracorporeal CPB circuit (e.g. TLR4) with signals that execute and facilitate the systemic inflammatory response (e.g. IL-6 or MMP9). Thus, the molecular signalling that underlies tissue ischemia and the inflammatory response seem to be highly intertwined. Intriguingly, the gene network analysis revealed a hierarchy in the transcriptional response, with key sensors of tissue hypoxia, such as HIF1alpha constituting the *hub nodes* connected with a number of downstream pro- and anti-inflammatory, i.e. *survival genes*. Our data indicate a critical role for tissue hypoxia as the key stimulus for the inflammatory response after cardiac surgery rather than other hitherto discussed mechanisms such as contact activation via CPB circuit or endotoxin exposure.

Interestingly, Emani et al. also reported HIF1alpha upregulation in peripheral blood transcriptome after CS/CPB. However, in that report the induction of HIF1alpha was detected selectively in patients with diabetes mellitus [22], an observation that is not supported by our data. Whole blood transcriptomics have further been employed to correlate gene regulation after CS/CPB with brain injury and artrial fibrillation [20], [21]. However, unsupervised direct hierarchical clustering of CS/CPB signatures did not clearly distinguish between patients with and without postoperative atrial fibrillation [21]. In contrast to our approach, dilution of target mRNA by the high content of globin mRNA (up to 70% of total mRNA) often limited the detection of low copy number genes leading to decreased sensitivity of the first generation whole blood transcriptomics data. Another explanation for the sensitivity of our approach to detect novel CS/CPB targets could be that we selected a rather homogenous study population consisting of predominantly elderly, hypertensive, and male individuals. This may have prevented additional biases induced by CS/CPB independent variables in microarray analyses.

In support of our data, Tomic et al. recently employed a 3.7 k tailored inflammation and immune response gene microarray and showed that key inflammatory cytokines (e.g. IL-6) and genes known to be involved in *priming* leukocytes such as adhesion molecules and toll-like receptors, were also upregulated following off-pump CABG to a similar extent as after CS/CPB, but with altered kinetics, i.e., with delayed onset [24]. These authors postulated that the current concept of contact activation by the extracorporeal CPB circuit and subsequent release of cytokines of circulating leukocytes might be oversimplified. Emerging data suggest that indeed the key components of the inflammatory response are also activated after off-pump surgery [24], [31]–[33]. However, since all participants in our study underwent CPB, we are unable to estimate the contribution of CPB to the inflammatory response observed following CS/CPB.

Intriguingly, we found a number of novel CS/CPB–induced genes such as *PTX3*, Toll-like receptors and *resistin* that were only recently linked to cardiac pathology, in particular myocardial infarction. One plausible explanation for our finding might be the transient cardiac ischemia that occurs during cardiac surgery with CPB, which parallels the pathophysiology of myocardial infarction. Of note, the multi-center study of the Perioperative Ischemia Research Group reported that up to 25% of patients fulfilled electrocardiogram, cardiac marker or autopsy criteria for myocardial infarction after CABG [34]. Therefore, the CS/CPB-induced pathways identified in the present report might have important implications for a better understanding of the pathophysiology and identification of novel biomarkers of other myocardial ischemia related disorders such as myocardial infarction.

Pentraxin 3 was recently considered as a master crossroad of innate immunity and the inflammatory response. Leukocytes (i.e., phagocytes) were described as the key source of this fluid-phase pattern recognition receptor (PRR). This might be a plausible explanation for the ability of our approach to detect this gene by genome-wide analysis of circulating blood leukocytes. In alignment with our data, PTX3 plasma concentrations were shown to be increased in patients with acute myocardial infarction and were therefore suggested as an early indicator for infarction and irreversible injury of the myocyte in ischemic cardiomyopathy [35]. Likewise, Suzuki et al. identified plasma PTX3 as a novel independent predictor of the clinical outcome in patients with heart failure [36]. Most recently, Salio et al. discovered a cardioprotective function for *PTX3* in acute myocardial infarction experimentally induced by coronary artery ligation and reperfusion [37]. They found greater than 30% increased myocardial damage in *PTX3*-deficient *vs.* wild type mice, which was accompanied by enhanced leukocyte infiltration, no-reflow areas, complement C3 deposition in the lesions, and apoptosis of the cardiomyocytes, while the number of capillaries were decreased. Most importantly, the substitution of mice with exogenous PTX3 reversed the exacerbated heart damage in the *PTX3*-deficient mice, hence a potential non-redundant cardioprotective role for this protein. These recent studies suggest unique functional properties of PTX3 in the pathogenesis of cardiovascular diseases. Together with our data, PTX3 seems to be a very promising therapeutic target and valuable biomarker for cardiac pathophysiology.

We also found differential regulation of Toll-like receptors, more specifically *TLR4* and *TLR6*, another family of proteins that play a key role in the innate immune system and like *PTX3* belong to the PRR. Unlike Pentraxin, *TLR4* has been reported to serve a pro-inflammatory role in murine myocardial ischemia-reperfusion injury. TLR4-deficient mice sustained smaller infarctions and exhibited less inflammation compared to wild type mice after myocardial ischemia-reperfusion injury induced by 1 hour of coronary ligation followed by 24 hours of reperfusion [38]. This effect was assumed to be unrelated to the microbial pathogen or endotoxin–induced *TLR4* activation [38]. These data are in alignment with our observations of elevated *TLR4* mRNA expression levels in circulating human blood cells after CS/CPB and suggest a critical pro-inflammatory role for *TLR4* in contact activation of leucocytes via the extracorporeal membrane and myocardial ischemia-reperfusion injury, in addition to its role in innate immune response.

In addition, phosphoglycerate kinase 1 (PKG1) gene expression in response to CS/CPB was found in our patients. The protein encoded by this gene is a glycolytic enzyme that catalyzes the conversion of 1,3-diphosphoglycerate to 3-phosphoglycerate. In line with our data, induction of HIF1alpha appears to upregulate PKG1 expression [39]. PKG1 in turn induces CXCR4 expression, whose ligand, CXCL12, also known as stromal derived factor-1 (SDF-1), was shown to be induced by tissue hypoxia. The activation of SDF-1-CXCR4 plays a pivotal role in regenerative mechanisms following hypoxic tissue damage for example in brain [40], heart [41] and kidney [42], including homing of pluripotent regenerative stem cells to sites of ischemic tissue injury [43]. Therefore, activation of the HIF1alpha-PKG1 axis might describe another possible mechanism for the regenerative tissue response induced by CS/CPB. The CS/CPB-induced upregulation of *resistin* in our patients also provides an interesting link between ischemia reperfusion injury and the inflammatory response. In 2001, Steppan et al. discovered a hormone that was suggested to link obesity with type 2 diabetes mellitus [44]. It was postulated that this protein secreted by mouse adipocytes might confer resistance to insulin and was therefore named *resistin* [44]. However, later studies in patients with renal disease found a correlation between elevated blood *resistin* levels and inflammation or decreased glomerular filtration rate but not with insulin resistance [45]. Moreover, *resistin* was shown to be primarily expressed in human inflammatory cells (in contrast to rodents) and to disturb the function of polymorphonuclear leukocytes, e.g. via impairment of their chemotactic movement, thus, contributing to the pathologic immune response associated with chronic kidney disease or diabetes mellitus [46], [47]. These data support our finding of elevated *resistin* mRNA expression in circulating blood leukocytes. Recently, it was reported that resistin-induced proinflammatory effects on the vascular wall might be triggered by hypoxia [48]. Hence differential regulation of *resistin* following CS/CPB might provide another link between tissue ischemia and the inflammatory response. In this context, high plasma *resistin* levels were found to predict mortality in patients with acute myocardial infarction and to worsen cardiac ischemia-reperfusion injury via impaired contractile recovery during reperfusion [49], [50]. Together, the herein newly identified CS/CPB-induced genes such as *PTX3*, Toll-like receptors and *resistin* may represent interesting targets for the development of CS/CPB-related biomarkers and/or therapeutic strategies.

We have recently shown that transcriptome analysis provides a powerful tool to uncover the molecular participants of homeostatic processes, such as angiogenic balance and the angiogenic switch [26], [27]. Integrative analysis of our whole blood transcriptomics approach suggests that the pro-inflammatory response evoked by cardiac surgery with CPB is also balanced by anti-inflammatory and pro-survival response elements. For example among the three abovementioned CS/CPB-induced pathways *resistin* and *TLR4* exert pro-inflammatory effects whereas *PTX3* exhibit cardioprotective effects. This balanced response may therefore protect organs from ischemia reperfusion-induced injury. By contrast, an imbalanced inflammatory response may herald the onset of the SIRS and organ dysfunction. Whether the here identified genes could assist clinicians to predict the inflammatory status of individuals at risk or aid in development of novel strategies to revert the systems imbalance by developing targeted therapies remains to be elucidated in prospective studies.

## Supporting Information

Table S1 SOM Table S1
(0.87 MB XLS)

Appendix S1 (0.03 MB DOC)
