# Supplementary material for: Whole Blood Transcriptomics in Cardiac Surgery Identifies a Gene Regulatory Network Connecting Ischemia Reperfusion with Systemic Inflammation
Source: PLoS One. 2010 Oct 27;5(10):e13658. doi: 10.1371/journal.pone.0013658 (PMC2965092; doi:10.1371/journal.pone.0013658)
Supplement: Appendix S1 — (0.03 MB DOC) [file pone.0013658.s002.doc]

**Appendix 1**

**Hepatocyte Growth Factor:**

TaqMan(R) Gene Expression Assays

Assay ID: Hs00300159_m1

**Hepatocyte Growth Factor Receptor:**

TaqMan(R) Gene Expression Assays

Assay ID: Hs01565580_m1

**Hypoxia-inducible factor 1, alpha subunit:**

TaqMan(R) Gene Expression Assays

Assay ID: Hs00936368_m1

**Matrix metallopeptidase 9:**

TaqMan(R) Gene Expression Assays

Assay ID: Hs00234579_m1

**Interleukin 18 receptor 1:**

TaqMan(R) Gene Expression Assays

Assay ID: Hs00977691_m1

**Interleukin 18:**

TaqMan(R) Gene Expression Assays

Assay ID: Hs01038788_m1

**CCAAT/enhancer binding protein (C/EBP), beta:**

TaqMan(R) Gene Expression Assays

Assay ID: Hs00942496_s1

**Phosphoglycerate kinase 1:**

TaqMan(R) Gene Expression Assays

Assay ID: Hs99999906_m1

**Glyceraldehyde-3-phosphate dehydrogenase:**

TaqMan(R) Gene Expression Assays, Inventoried

Assay ID: Hs99999905_m1

**Lipocalin 2:**

TaqMan(R) Gene Expression Assays

Assay ID: Hs00194353_m1

**Resistin:**

TaqMan(R) Gene Expression Assays

Assay ID: Hs00220767_m1

**Toll-like receptor 4**

TaqMan(R) Gene Expression Assays

Assay ID: Hs01060206_m1

**Pentraxin-related gene, rapidly induced by IL-1 beta:**

TaqMan(R) Gene Expression Assays

Assay ID: Hs00173615_m1

**18 S ribosomal RNA:**

TaqMan(R) Gene Expression Assays

18 S Assay Id: Hs99999901_s1

**Beta-2-macroglobulin:**

TaqMan(R) Gene Expression Assays

B2M Assay Id: Hs99999907_m1
